# Spatial modeling and ecological suitability of monkeypox disease in Southern Nigeria

**DOI:** 10.1371/journal.pone.0274325

**Published:** 2022-09-20

**Authors:** Temitope Emmanuel Arotolu, Ayoola Ebenezer Afe, HaoNing Wang, JiaNing Lv, Kun Shi, LiYa Huang, XiaoLong Wang

**Affiliations:** 1 Center of Conservation Medicine & Ecological Safety, Northeast Forestry University, Harbin, Heilongjiang Province, P. R. China; 2 Key Laboratory of Wildlife Diseases and Biosecurity Management, Harbin, Heilongjiang Province, P. R. China; 3 College of Wildlife and Protected Area, Northeast Forestry University, Harbin, Heilongjiang Province, P. R. China; 4 Animal Genetic Engineering and Geoplasm Innovation, Graduate School of Chinese Academy of Agricultural Sciences, Beijing, P. R. China; 5 School of Geography and Tourism, Harbin University, Harbin, Heilongjiang Province, P. R. China; 6 Wildlife Institute, Beijing Forestry University, Beijing, Beijing, P. R. China; 7 Changbai Mountain Academy of Sciences, Antu, Jilin Province, P. R. China; The University of Hong Kong, CHINA

## Abstract

The reemergence of monkeypoxvirus (MPXV) in 2017 after about 39 years of no reported cases in Nigeria, and the recent incidence in countries such as the United States of America, United Kingdom, Singapore, and Israel which have been reportedly linked with travelers from Africa, have heightened concern that MPXV may have emerged to occupy the vacant ecological and immunological niche created by the extinct smallpox virus. This study was carried out to identify environmental conditions and areas that are environmentally suitable (risky areas) for MPXV in southern Nigeria. One hundred and sixteen (116) spatially unique MPXV occurrence data from 2017–2021 and corresponding environmental variables were spatially modeled by a maximum entropy algorithm to evaluate the contribution of the variables to the distribution of the viral disease. A variance inflation analysis was adopted to limit the number of environmental variables and minimize multicollinearity. The five variables that contributed to the suitability model for MPXV disease are precipitation of driest quarter (47%), elevation (26%), human population density (17%), minimum temperature in December (7%), and maximum temperature in March (3%). For validation, our model had a high AUC value of 0.92 and standard deviation of 0.009 indicating that it had excellent ability to predict the suitable areas for monkeypox disease. Categorized risk classes across southern states was also identified. A total of eight states were predicted to be at high risk of monkeypox outbreak occurrence. These findings can guide policymakers in resources allocation and distribution to effectively implement targeted control measures for MPXV outbreaks in southern Nigeria.

## Introduction

Monkeypox, a re-emergent viral zoonotic disease of public health importance in Nigeria, is a member of the genus Orthopoxviridae, which can cause a serious, smallpox-like illness in humans [1]. Monkeypox occurs primarily in tropical rainforest areas of Central and West Africa and is occasionally exported to other regions [2]. Since the global eradication of smallpox in 1977, the monkeypox virus (MPXV) has been considered the most problematic orthopoxvirus as regards human health [3]. Human monkeypox is endemic to forested areas of West and Central Africa and is thought to be transmitted to humans through contact with infected animals and through person-to-person contact [4]. Monkeypox can infect a taxonomically wide range of mammalian species, but the natural host is unknown. The virus has only been isolated twice from a wild animal, a rope squirrel (*Funisciurus sp*.) in the Democratic Republic of Congo (DRC) [5], and a sooty mangabey (*Cercocebus atys*) in the Ivory Coast [6].

In an African setting, monkeypox can be misdiagnosed as other rash illnesses. The most common misdiagnosis (up to 50%) of suspected monkeypox cases in the DRC [4] is chickenpox, also known as varicella, caused by the varicella-zoster virus. Coinfections of both MPXV and varicella-zoster virus has been reported only a few times [7]; however, it has been recently suggested that this is a relatively common phenomenon [8]. The role of the varicella-zoster virus in MPXV epidemiology is not clear. Besides chickenpox, monkeypox can be misdiagnosed as cutaneous anthrax, fungal infection in Human Immunodeficiency Virus (HIV) patients, Staphylococcus sp. related rash [9], or other diseases which cause a rash.

The first MPXV index case in Nigeria was recorded in 1971, and 10 MPXV cases were reported between 1971 and 1978 [10]. Since then, several thousand human cases of monkeypox have been confirmed in 15 different countries, with 11 of them in African countries [11]. In Nigeria, as at 31^st^ october, 2021, monkeypox cases have been recorded in 26 states out of the 36 states of the country (the Federal Capital Territory inclusive), an increase to the 11 states initially reported in 2017 [12, 13]. According to the World Health Organization (WHO), MPX is commonly found in young persons below the age of 40 or 50 (varies with country) caused by the cessation of smallpox vaccination after the eradication of smallpox in 1980 [2]. The WHO report reveals that most monkeypox cases occur among persons under the age of 40, with a median age of 31 years [14]. Asides from the age distribution, the gender distribution of the disease has also been studied confirming that the male gender is more affected than the female [15].

The two possible means of MPXV transmission are animal–human transmission and human-human transmission. Respiratory droplets and contact with body fluids, contaminated patient’s environment or items, and skin lesion exudate of an infected person have been found to be associated with inter-human transmission. Viral shedding via feces may represent another exposure source [16, 17]. Animal-to-human transmission, which is also known as zoonotic transmission, occurs via direct contact with any of the aforementioned natural viral hosts or consumption of these hosts. In addition, zoonotic transmission could occur by direct contact with the blood, body fluids, and inoculation from mucocutaneous lesions of an infected animal [17]. Nosocomial transmission has been reported [12, 18], while sexual transmission has been speculated for infected individuals with groin and genital lesions [19]. At present human-to-animal transmission has not been reported.

Studies have shown that vaccination against smallpox provides cross-protection against other Orthopoxvirus (OPV) species, including MPXV. According to the available data, about 90% of the identified cases are naive to OPV infection, of which many of them were born after the cessation of the smallpox eradication program [20]. Individuals who had previously been vaccinated against smallpox were identified to have 85% protection against MPXV [14, 20].

The frequency and geographical spread of monkeypox cases have increased in recent years, and there are huge gaps in our understanding of the disease’s emergence, epidemiology, and ecology. Therefore, this study uses ecological niche modeling techniques in seeking an understanding of suitable MPX habitat based on the environmental variables of known occurrence sites [21]. Maximum entropy (MaxEnt) model is regarded as one of the best-performing species distribution modeling techniques for analyzing presence-only data [22]. It creates ecological niche models by combining presence-only data with environmental variables using a machine-learning approach known as maximum entropy. The reliability of MaxEnt has been confirmed by its good capacity to predict novel presence localities for poorly known species/diseases [23]. Therefore, this study aims to use the MaxEnt algorithm model to understand the distribution pattern and risk areas in southern Nigeria and to produce epidemiological evidence to guide the management of MPXV disease outbreaks in Nigeria.

## Materials and methods

### Study area

**Fig 1:**
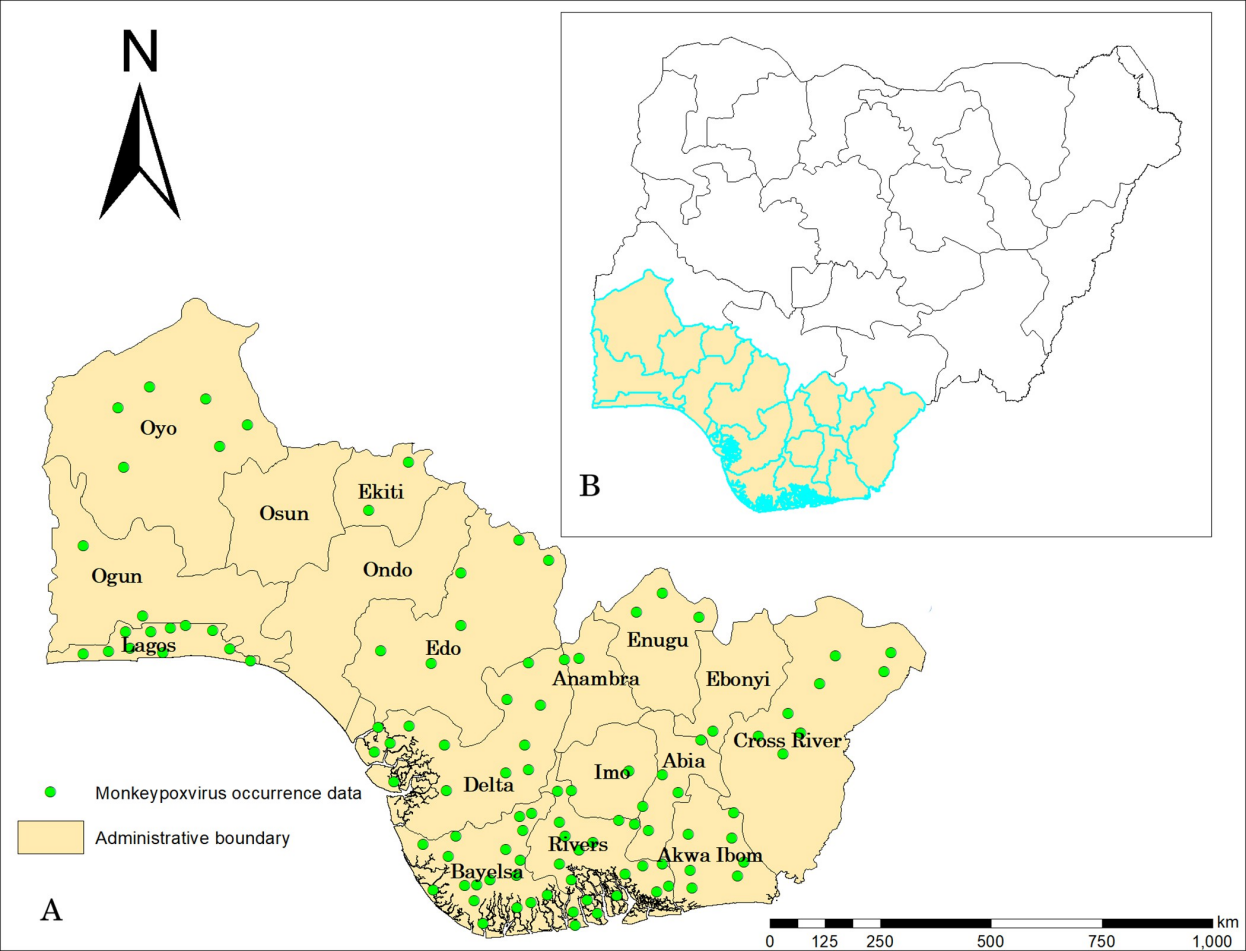
Study Area: A–Southern Nigeria; B–Nigeria (study area in red).

Our study was limited to the southern part of Nigeria (Fig 1), as about 94% of the confirmed cases were within this area. Only 12 out of 199 confirmed monkeypox cases by state, September 2017- September 2021 were from the northern part of Nigeria. Rainfall is often the key climatic variable, and there is a marked distinction of wet and dry seasons in most areas. Two air masses control rainfall-moist northward-moving maritime air coming from the Atlantic Ocean and dry continental air coming south from the African landmass. The climate becomes drier as one moves north from the coast. The estimated human population in southern Nigeria was 91,442,452 in 2021 [24].

### MPV occurrence data collection and preprocessing

There were 199 recorded MPX disease outbreak locations (S1 Table) collected from the Nigeria Center for Disease Control reports from 2017–2021 [25]. We filtered the presence points using the SDM Toolbox v1.1c [26] integrated in ArcGIS 10.6 to minimize potential spatial autocorrelation [27]. Filtering was performed by limiting the minimum distance between each pair of occurrence points at 10 km [28, 29]. This helped to address problems associated with spatial sampling biases. Ideally, filtering or thinning removes the fewest records necessary to substantially reduce the effects of sampling bias, while simultaneously retaining the occurrence points with the greatest amount of useful information [30, 31]. The geo-coordinates were then saved in a comma-separated value (CSV) format and imported into ArcGIS (version 10.6 ESRI) for editing and projected in UTM-WGS-1984 with standard settings or resampling to 30 arc-seconds.

### Environmental data preprocessing

A total of 68 climatic and human population density variables (S2 Table) were used which was extracted from the WorldClim version 1.4 dataset from 1950–2000 at 30 arc–second resolution [32] and worldpop dataset available at www.worldpop.org. WorldPop dataset are available to download in Geotiff and ASCII XYZ format at a resolution of 30 arc-seconds. Also, it is the unconstrained individual countries 2000–2020 population count datasets. We explored the existence of multicollinearity using the variance inflation factor (VIF). Multicollinearity often arises in statistical models, and can be a severe problem for parameter estimation because it inflates the variance of regression parameters and hence potentially leads to the wrong identification of relevant predictors in an ecological model [33]. The Variance inflation factor represents the amount of variability of a covariate which is explained by other covariates. A VIF >10 has been considered to indicate highly correlated variables which are then typically removed from the input data set [34]. Of the 68 covariates initially tested for multicollinearity, thirty-six (36) were excluded from further analysis. All remaining covariates were considered to be independent and were included in the analysis. The multicollinearity test was implemented using the statistical package for social sciences (spss v22.0). Furthermore, the remaining bioclimatic variables, human population density and elevation were subsequently processed in MaxEnt algorithm, three methods; Jackknife test, backward stepwise variable elimination, and the variable response curves were selected to identify the relative contribution of predictor variables to the model. Variables with a percentage contribution less than one percent were removed from the model.

### Model development and evaluation

A MaxEnt model was fitted using 100 bootstrap runs and a 70/30 partition percentage for the training/testing datasets. The advanced options in MaxEnt that were selected include the maximum iteration set to 5000 to allow the models to have enough time to reach convergence at 0.00001 [35], and 90% sensitivity was set within the MaxEnt model for determining suitability. The area under the Receiver Operating Characteristics [36] was used to assess the accuracy of the model. In the MaxEnt model, the Area Under the Curve (AUC) of the receiver operating characteristic plot was used as an evaluation criterion to assess the accuracy of the model [37]. We reclassified the MaxEnt spatial model output into three risk classes, namely high, moderate and low risk areas.

## Results

**Fig 2:**
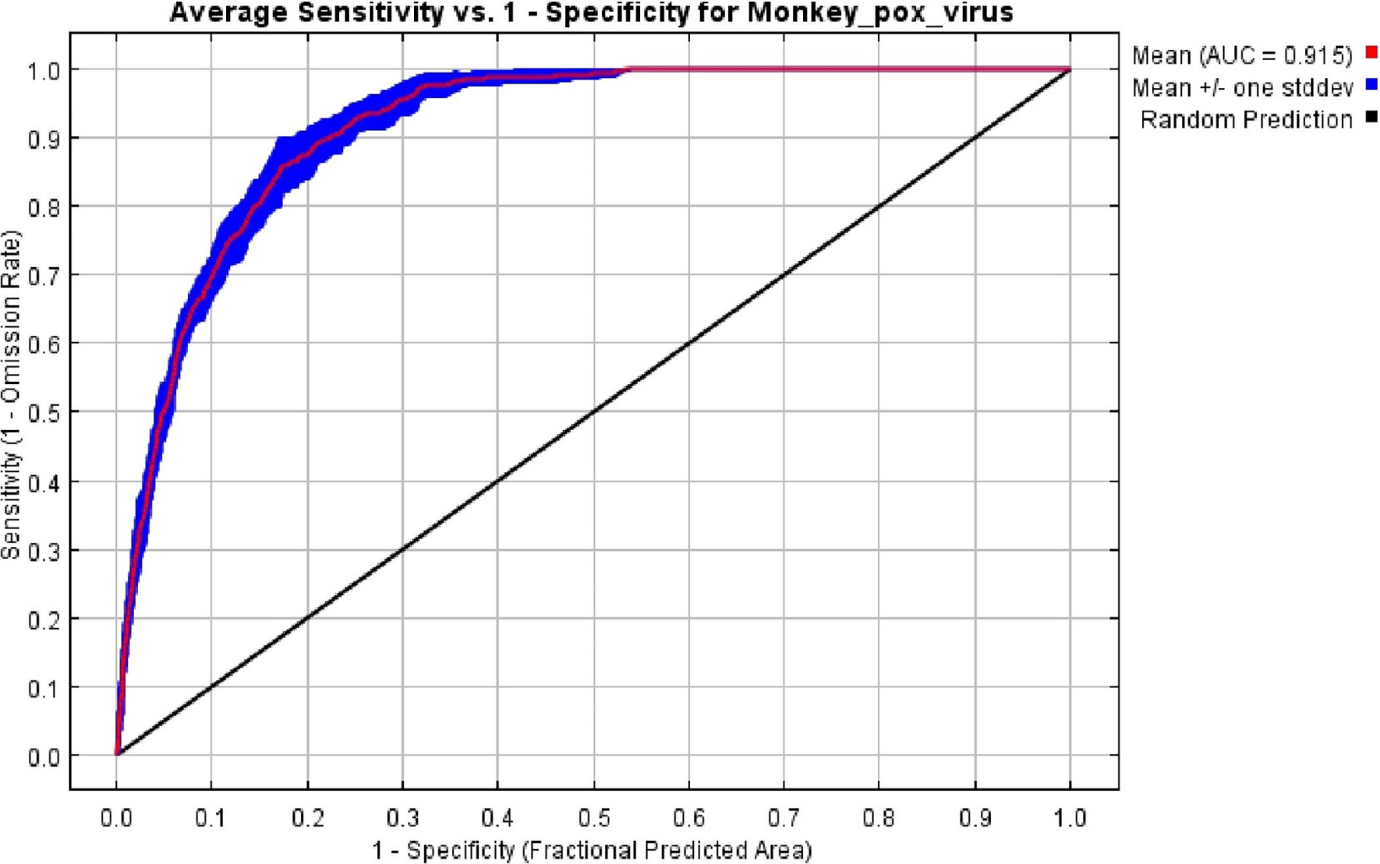
Average receiver operating characteristics [36] and related area under the curve (AUC) of the 100 bootstrap replicates.

**Fig 3:**
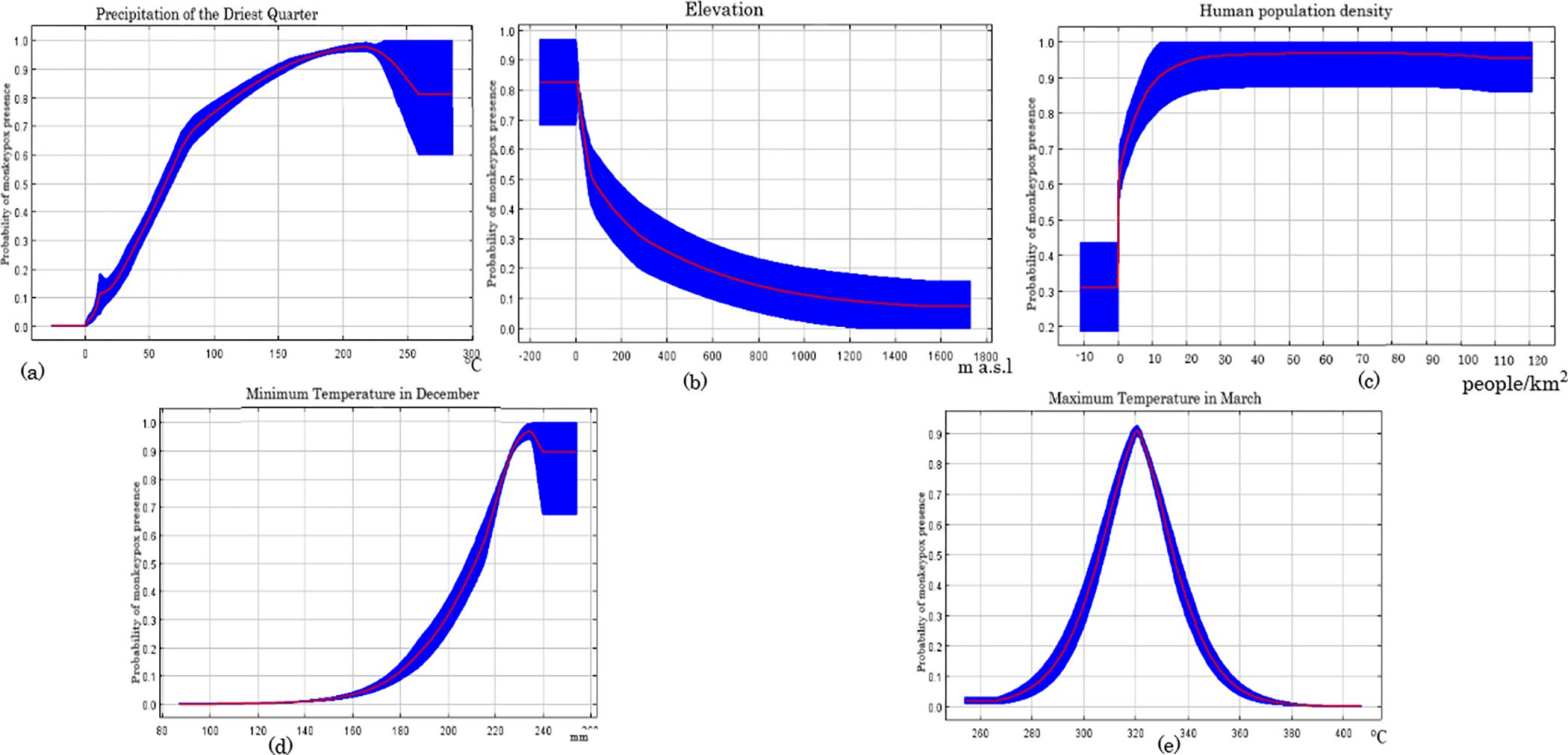
Response curves of predictive continuous variables ((a) precipitation of driest quarter, (b) elevation, (c) human population density, (d) minimum temperature in December, and (e) maximum temperature in March) for monkey pox suitability in southern Nigeria. The red lines indicate the mean values while the blue lines denote the standard deviation.

The spatial rarefication of occurrence data selected 116 out of 199 present records at a distance of at least 10km away from each other, after duplicates and ouliers points were removed. VIF analysis filtered the 69 environmental variables to 32 independent variables. The independent variables were fitted in the modeling process. After the occurrence data filtering, VIF, and MaxEnt filtering, precipitation of the driest quarter, elevation, human population density, minimum temperature in December, and maximum temperature in March predictor were the predictor variables which remained (Table 1). For validation, our model had a high AUC value of 0.92 and a standard deviation of 0.009, indicating that it had an excellent ability to predict the suitability areas for monkeypox disease (Fig 2). Five variables contributed >1%, namely, precipitation of driest quarter (47%), elevation (26%), human population density (17%), minimum temperature in December (7%), and maximum temperature in March (3%) (Table 1). The response curves of the predictors show that the precipitation of the driest quarter (15–25 mm), elevation (800m), human population density (10 people/km^2^), minimum temperature in December (22°C above), and the maximum temperature in March (32°C) can influence MPX (Fig 3).

**Table 1:** Contribution of the five environmental predictors to the final suitability model.

Variable	Percentage contribution	Permutation importance	Unit
Precipitation of driest quarter	47	37	Milimeter (mm)
Elevation	26	33	Meters above sea level (m. a. s. l)
Human population density	17	10	People/km^2^
Minimum temperature in December	7	9	Degrees celcius (°C)
Maximum temperature in March	3	11	Degrees celcius (°C)

**Fig 4:**
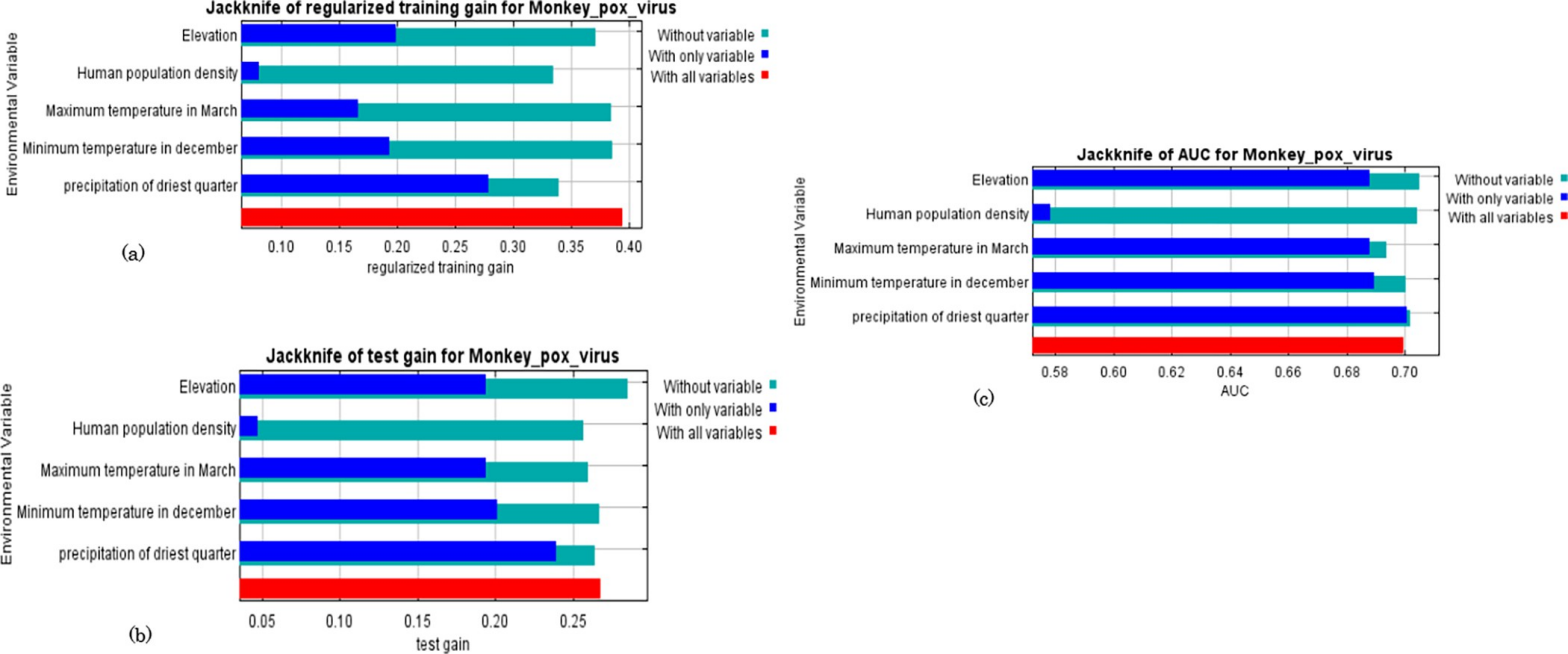
Summary of the Jackknife analysis performed to determine importance of each environmental variable.

The Jackknife test of variables shows that omitting any of these five variables affects the regularization gain, test gain, and AUC in the model (Fig 4). Precipitation of the driest quarter had the highest training gain when each variable was tested as the only environmental variable, and the lowest values were observed in the human population density. The lowest training gain appeared when the human population density was excluded from the model, while the model had the highest gain when minimum temperature in December and maximum temperature in the March were excluded (Fig 4). Precipitation of the driest quarter had the highest test gain values when used as the only environmental variable, and human population density has the least test gain among the variables.

**Fig 5:**
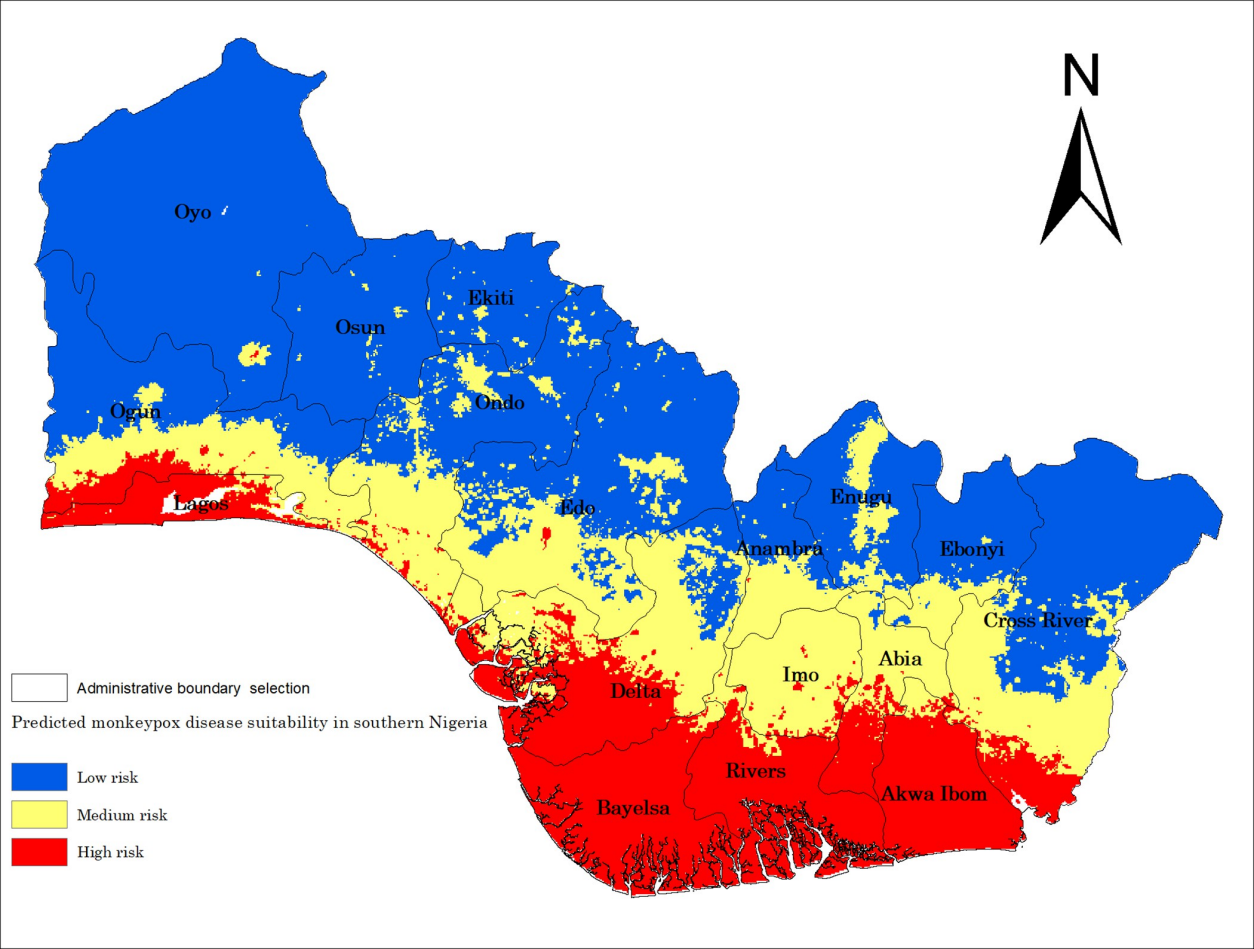
Ecological suitability map of monkeypox disease in southern Nigeria.

MPX disease high-risk areas in southern Nigeria were predicted using the MaxEnt algorithm model (Fig 5). High-risk areas were distributed in Lagos, Delta, Bayelsa, Rivers, Akwa Ibom, Imo, Abia, and cross River. Additionally, MPX disease high-risk areas were scattered throughout Ondo Anambra and Enugu state. Medium risk areas are Ogun, Ondo, Edo, Anambra, and Enugu. Potentially low-risk areas are Oyo, Osun, Ekiti, Anambra, and Ebonyi. It is worth noting that one, high-risk areas show an obvious trend of distribution along the Atlantic Ocean, and two, the risk decreases as the latitude increases from the south toward the north.

## Discussion

### MPX risk and variable analysis

In this study, an ecological niche modeling technique was used to predict the potential suitable habitat distribution of the monkeypox virus in southern Nigeria. Our study presents the first risk assessment of ecologically suitable areas for monkeypox disease in Nigeria. Precipitation of the driest quarter (15-25mm) is the most important predictive variable in our model, with a contribution of 47%. Other study have shown that monkeypox disease outbreaks are associated with precipitation as the chief environmental predictor in the model [38]. Also, during the comparison between the Central and West African monkeypox forms, it was observed that monkeypox in West Africa occurs in a wetter and hotter condition which is a subtle difference in ecological condition to its Central African form [38]. The second most important predictive variable is elevation (below 800 m. a.s.l). The relatively low elevation in the south could be the primary reason for a higher number of monkeypox cases when compared to the northern counterpart. Our model predicted human population density as an important variable in monkeypox distribution, which may be caused by increased human-environment interaction. Minimum temperature in December (above 22°C) and maximum temperature in March (32°C) are the least predictive variable in our model. Temperature has been suggested to play an important role in transmitting and spreading infectious diseases [39]. However, the response curves remind us that the appropriate temperature (minimum temperature in December = above 22°C) and (maximum temperature in March = 32°C) can increase the risk of MPX disease. These alert us to pay close attention to temperature and precipitation seasonality in preventing MPX in Nigeria. Our study reveals that climatic factors and elevation are fundamental conditions that influence MPXV distribution in southern Nigeria. Also, human population density and other related human activities are specific factors reshaping the spatial distribution of MPXV. The predicted variables don’t translate to singly influencing the disease distribution or suitability. Some variables might act in proxy or directly influencing the host abundance, behaviour or activities. Two or more variables might jointly act to foster disease outbreak.

### Ecological suitability of monkeypox disease

Our model predicted areas with suitable environmental conditions for MPXV around southern Nigeria. The high-risk area covered the southwest, southeast and south-south part of the region, which include Lagos, Delta, Bayelsa, Rivers, Imo, Abia, Akwa Ibom, and Cross River.

The predicted geographic distribution of MPX defines some suitable areas with no historical outbreak records in Ondo, Osun, and Ebonyi state (only one confirmed case), which may either be over-prediction of the model or poor surveillance systems, leading to severe under-reporting and hence misleading disease burden information. However, it is important to note that over-predicting the geographic distribution of a species does not necessarily infer prediction error. The potentially over-predicted geographical distribution areas may represent an accurate illustration of the spatial disease extent [40], despite the lack of presence records that could be used for testng the accuracy of our model in those states.

### Population at risk

Monkeypox has a low fatality rate (0–11%), with the highest rates occurring in children and young persons who were not part of the population vaccinated against smallpox. Previous study once suggests that consumption of bush meats could be a potential risk factor in the transmission of the infection, as could caring for an infected patient [15]. A new vaccine was produced in 2019 for the prevention of monkeypox. However, there is an ongoing study to validate the effectiveness and safety of the Modified Vaccinia Ankara (MVA) vaccine in human cases as it is believed to confer immunity against all orthopoxviruses [41, 42].

## Conclusions

In our study, a MaxEnt model was used to predict the potential distribution of MPX disease in southern Nigeria. Predicted environmental variables in our model give insight into the time and season when surveillance efforts should be intensified to combat MPX outbreaks. Similarly, our risk map with categorized risk, which helps in grading states into high, moderate, and low-risk areas, could guide in resources allocation and distribution, with special attention given to children and young persons who were not part of the smallpox vaccination population.

## Supporting information

S1 Table The record of monkeypoxvirus outbreak with latitude and longitude information of the location. (XLS)

S2 Table Summary of variables used for initial modeling in MaxEnt software. (DOC)
