# Supplementary material for: Spatial modeling and ecological suitability of monkeypox disease in Southern Nigeria
Source: PLoS One. 2022 Sep 20;17(9):e0274325. doi: 10.1371/journal.pone.0274325 (PMC9488772; doi:10.1371/journal.pone.0274325)
Supplement: S2 Table — (DOC) [file pone.0274325.s002.doc]

**S2 Table:** **Summary of variables used for initial modeling in MaxEnt software.**

| **Class** | **Variable** | **Source** |
| --- | --- | --- |
| Bioclimatic variables | Annual Mean T**^†^**, Mean Monthly Diurnal Range (Tmax - Tmin), Isothermally (Mean Monthly Diurnal Range / T Annual Range) x 100, T Seasonality (Standard Deviation), Max T of Warmest Month, Min T of Coldest Month, T Annual Range (T Seasonality - Max T of Warmest Month), Mean T of Wettest Quarter**^†^**, Mean T of Driest Quarter, Mean T of Warmest Quarter, Mean T of Coldest Quarter, Annual P**^†^**, P of Wettest Month**^†^**, P of Driest Month, P Seasonality (Coefficient of Variation), P of Wettest Quarter**^†^**, P of Driest Quarter, P of Warmest Quarter, P of Coldest Quarter**^†^** | Worldclim^a^ |
| Elevation | Elevation | Worldclim^a^ |
| Classical meteorological variables | January precipitation, February precipitation**^†^**, March precipitation**^†^**, April precipitation**^†^**, May precipitation**^†^**, June precipitation, July precipitation**^†^**, August precipitation**^†^**, September precipitation**^†^**, October precipitation**^†^**, November precipitation**^†^**, December precipitation, Minimum temperature in April**^†^**, Minimum temperature in May**^†^**, Minimum temperature in June**^†^**, Minimum temperature in July**^†^**, Minimum temperature in August, Minimum temperature in September**^†^**, Minimum temperature in October, Minimum temperature in November**^†^**, Minimum temperature in December, Mean temperature in January, Mean temperature in February**^†^**, Mean temperature in March**^†^**, Mean temperature in April**^†^**, Mean temperature in May, Mean temperature in June**^†^**, Mean temperature in July, Mean temperature in August, Mean temperature in September**^†^**, Mean temperature in October**^†^**, Mean temperature in November, Mean temperature in December, Maximum temperature in January**^†^**, Maximum temperature in February**^†^**, Maximum temperature in March, Maximum temperature in January**^†^**, Maximum temperature in February**^†^**, Maximum temperature in March, Maximum temperature in April, Maximum temperature in May, Maximum temperature in June, Maximum temperature in July**^†^**, Maximum temperature in August**^†^**, Maximum temperature in September, Maximum temperature in October**^†^,** Maximum temperature in November**^†^**, Maximum temperature in December**^†^** | Worldclim^a^ |
| Human impact | Human population density | WorldPop^b^ |
| **^a=^ T:** Temperature; **P:** precipitation; source: [www.worldclim.org/](http://www.worldclim.org/) **^b=^** Source: [www.worldpop.org/](http://www.worldpop.org/) **^†=^** The 36 variables excluded in the modeling process. | |  |
